# Facile Control of a Tailed Virus Surrogate by Iron Conventional Coagulation and Electrocoagulation

**DOI:** 10.1021/acs.est.5c02792

**Published:** 2025-07-30

**Authors:** Kyungho Kim, Anindito Sen, Shankararaman Chellam

**Affiliations:** † Department of Civil & Environmental Engineering, Texas A&M University, College Station, Texas 77843-3136, United States; ‡ Microscopy and Imaging Center, Texas A&M University, College Station, Texas 77843-2257, United States; § Department of Chemical Engineering, Texas A&M University, College Station, Texas 77843-3122, United States

**Keywords:** Cryogenic electron microscopy, water treatment, wastewater treatment, potable reuse, bacteriophage

## Abstract

Both FeCl_3_ conventional coagulation and Fe(0) electrocoagulation were highly effective in mitigating the long-tailed somatic phage P1. We targeted enterobacterial coliphages because they are better than fecal indicator bacteria in tracking environmental persistence of viral pathogens and their fate in wastewater unit operations. Cryogenic electron microscopy (cryo-EM) of intact/damaged P1 and enumeration of infective virions by plaque assays demonstrated control via both removal and inactivation. Cryo-single particle analysis was coupled with cryo-electron tomography to generate 3-dimensional electron density maps to visualize and analyze untreated and coagulated capsids. To our knowledge, this is the first report of cryo-EM to visualize structurally damaged viruses with environmental relevance. Viruses were intrinsically enmeshed in precipitates consistent with sweep coagulation. Direct evidence of multiple inactivation mechanisms was obtained including (i) capsid breakage leading to leakage of viral genome and other components, (ii) deformation and thinning of capsid proteins, (iii) severance/damages to the neck region where the tail is attached to the capsid, (iv) removal, fragmentation, and splintering of tail sections, and (v) baseplate damage (including receptor-binding proteins). Conformational alterations to proteins, changes to secondary structures, and specific interactions with flocs were inferred from infrared spectroscopy for both coagulation approaches. However, only electrocoagulation oxidized proteins. Extremely facile reduction of P1 suggested that coliphages with myovirus morphology may not be conservative surrogates to measure log reduction values for regulatory purposes and public health protection by iron conventional coagulation and electrocoagulation.

## Graphical abstract

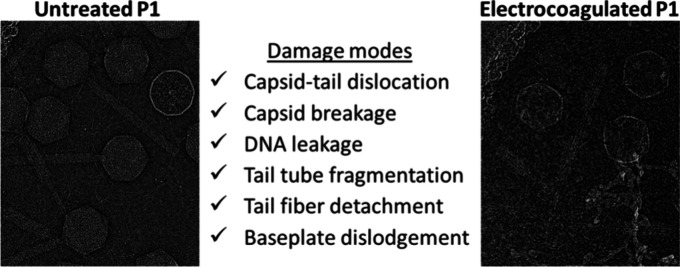

## 1 Introduction

It has been reported that annually ∼7 million cases of waterborne illnesses occur in the United States costing >$3 billion,^1^ which are magnified severalfold worldwide.^2^ Viruses shoulder a portion of these health/financial burdens because they are infectious at low concentrations, can survive for extended periods in the aquatic environment, often exist asymptomatically for prolonged timeframes in their hosts, and are excreted in large numbers.^3,4^ Indeed, they have been implicated in acute gastroenteritis and other waterborne illnesses and are the leading etiologic agent of infant diarrhea in developing countries.^5,6^ Therefore, virus control is central to drinking water safety,^6^ which has taken on added significance given the increasing implementation of potable reuse globally.^7–9^ One of the main drivers of our research is the stringent virus log reduction values (LRVs) that have been mandated for indirect and direct potable reuse (IPR and DPR, respectively). For example, the State of California has imposed virus LRVs of 12 and 20 for IPR^10^ and DPR,^11^ respectively and Australia mandates a 9.5 enteric virus LRV for IPR,^12^ all of which are substantially higher than the 4-logs requirement under United States’ Safe Drinking Water Act. One method of increasing cumulative LRVs of potable reuse treatment trains is adding unit processes for which coagulation is a prime candidate, given its ability to remove/inactivate viruses coupled with its long history in conventional treatment for colloid destabilization, disinfection byproduct precursor removal, and membrane pretreatment.^13–17^ Another research motivation is the growing interest in decentralizing water treatment infrastructure to increase resiliency,^18,19^ which requires small-scale units and potentially new technologies,^20^ for which electrocoagulation is a promising option.

Much of our existing knowledge on virus mitigation by water/wastewater unit operations is based on icosahedral/spherical phages or a human virus such as MS2, Qβ, ϕX174, PRD1, ϕ6, adenovirus, etc.^6,13,21–31^ On the other hand, municipal wastewaters contain high concentrations of tailed viruses,^32–35^ which could be stable even in harsh aqueous environments^36^ and might be more resistant to removal/inactivation using ozone,^37^ UV/H_2_O_2_,^38^ aluminum coagulation,^39^ zerovalent iron,^40^ or electrochemical treatment^41^ (Table S2 in the Supporting Information (SI)). Also, to date, all known human viruses are nontailed, ensuring an additional level of operator and laboratory safety and easy handling/disposal when working with tailed phages. Hence, tailed viruses are potentially excellent surrogates for evaluating process performance and LRVs especially in light of the multiple barrier approach to water treatment and potable reuse^42,43^ because they are (i) common in wastewater, (ii) nonpathogenic toward humans, and (iii) possibly reduced to a lower extent in selected unit processes. Therefore, we designed a study to evaluate LRVs of a tailed virus, conforming to calls from environmental virologists for the use of multiple surrogates and mechanistic studies of their reduction.^4,44^

Our primary objective was to mechanistically evaluate the removal and inactivation of a representative tailed phage (P1) during iron conventional coagulation and electrocoagulation. We imaged intact and damaged viruses using cryo-electron microscopy (cryo-EM), close to their native conditions, overcoming limitations inherent to room temperature transmission EM (TEM) such as fixing, dehydrating, and staining. Cryo-EM coupled with single particle analysis (SPA) and cryo-electron tomography (cryo-ET) have been employed to investigate virus’ fine structures^45–47^ and their spatial relation with the environment^48–50^ to reveal their functionality. In this work, electron density maps of intact virions were generated at 17 Å resolution by SPA and used to identify local capsid damage upon electrocoagulation. Also, cryo-EM images of electrocoagulated virions taken over a wide angular range were three-dimensionally (3-D) reconstructed, creating a tomogram at 55 Å resolution to unveil damages inflicted on virions and spatial interaction with iron flocs. Hence, wherever we interpreted tomograms, we rigorously reported only those damaged regions exceeding 55 Å. Identifying atomic-level modifications such as those to secondary protein structures via cryo-EM^51^ is beyond the scope of this study. SPA and cryo-ET results together provided a unique opportunity to reveal a comprehensive and detailed mechanistic picture of removal and inactivation by electrocoagulation in conjunction with infective viruses quantified by the plaque assay. To our knowledge, this is the first cryo-EM report of damaged virus structures generated to identify biophysical modifications leading to inactivation with relevance to environmental science/engineering. Biochemical modifications were probed with attenuated total reflection–Fourier transform infrared spectroscopy (ATR-FTIR).

## 2 Materials and Methods

### 2.1 Electrocoagulation and Conventional Coagulation

Like our recent work,^14,23,52^ all experiments were duplicated and performed in a 500 mL reactor using 3 mM NaHCO_3_ and 1 mM CaCl_2_ solution at pH 6.5 as the background electrolyte. Electrocoagulation was performed using 52.4 cm^2^ effective area low carbon steel (0.2%, McMaster-Carr, 99% purity as Fe) plate electrodes at a current density of 0.19 mA/cm^2^ using a power supply (Interface 1010E, Gamry Instruments). Initial experiments with dosages of 1 and 5 mg/L as Fe precipitously removed/inactivated P1 (e.g., LRV of ∼7 within only 3 min at 5 mg/L), making it nearly impossible to comprehensively measure its temporal profile. Therefore, a dose of only 0.2 mg/L Fe was chosen. The suspension was rapidly mixed during electrolysis (34.6 s to add 0.2 mg/L Fe), after which the electrodes were removed and slow-mixed (flocculated) for 1 h. Conventional coagulation was performed under the same conditions by adding 0.2 mg/L Fe of FeCl_3_·6H_2_O. Total iron concentrations were measured to be 0.21 ± 0.01 mg/L Fe in all cases.

### 2.2 Virus Infectivity Profiles

The enterobacterial phage P1 was propagated using MG1655 as the host following the confluent lysis method^53^ achieving a titer of ∼10^11^ PFU/mL^54^ (SI Section S1). This somatic coliphage was chosen because (i) coliphages with myovirus morphology are some of the most abundant viruses found in municipal wastewater and (ii) in general, coliphages more reliably track fecal contamination, environmental persistence of pathogenic viruses, and fate through treatment processes compared to indicator bacteria.^34,35^ During experiments, bulk water virus concentrations were measured at predetermined time intervals by pipetting 1 mL of suspension, syringe filtering (0.45 μm poly­(ether sulfone)), adding Na_2_SO_3_ (25 mM final concentration to quench H_2_O_2_ and dissolved oxygen to minimize potential downstream inactivation), and immediately performing a double agar assay.^55,56^ Total infective virus concentrations in the system (i.e., bulk water + flocs) were also measured after pipetting 100 μL of suspension using a wide-mouth pipet tip, dissolving precipitates in 900 μL of 6% beef extract eluent, and vortexing for 10 min.^14,39,57^ Transient profiles of infective fractions were obtained by normalizing the concentration at any given time (*N*_t_) by the initial concentration (*N*_0_ ≈ 10^7^ PFU/mL) based on at least four plates from duplicates, each displaying a minimum of 10 plaques. The phage detection limit was 10 PFU/mL for the bulk water and 100 PFU/mL for the total concentration (bulk water + flocs) based on the assay volume (1 mL and 100 μL, respectively). This is summarized in SI Section S2.

### 2.3 Conventional TEM

Untreated and electrocoagulated P1 phages were visualized using conventional TEM. The P1 stock was directly used for untreated phages. For electrocoagulated samples, the suspension was withdrawn at the end of electrocoagulation (*t* = 60 min), treated with 25 mM Na_2_SO_3_, and centrifuged (15000*g*, 24 h, and 4 °C). These centrifugation conditions effectively collected untreated P1 particles (SI Section S1). Hence, electrocoagulated phages in both bulk and flocs were also expected to be pelletized. After decanting the supernatant, the collected pellet was gently resuspended in 1 mL of TM buffer. Approximately 5 μL portions of samples were mounted on the carbon/copper 300-mesh grids supported on a Formvar layer prepared by glow-discharging for 1 min at 10 mA under vacuum (PELCO easyGlow) and negative stained using 2% uranyl acetate solution without adjusting pH. A FEI Tecnai G2 F20 TEM (TF20, Thermo Fisher Scientific) equipped with a Gatan K2 direct detector and a Gatan Tridiem GIF-CCD camera at a 200 keV accelerating voltage was used for image acquisition. After screening for a sufficient spatial population of phages on the grid and purity (for intact P1), a single batch of each sample (i.e., untreated and electrocoagulated phages) was imaged (see SI Section S3).

### 2.4 Cryo-EM, SPA, and Cryo-ET

Single batches of screened samples as described above were used for cryo-EM and downstream analysis. Our cryo-EM sample preparation followed the state-of-the-art procedures suitable for high-resolution biospecimen imaging and was designed to minimize damages to protein complexes.^51,58^ For untreated phages, cryo-EM imaging and SPA (for capsids only) were conducted as follows. Using a high-quality P1 stock, cryo-grids with phages entrapped in vitreous ice were prepared and inspected for the ice quality and P1 particle distribution. Afterward, the P1 stock was supplemented with 10 nm gold nanoparticles as fiducial markers. Three μL of this mixture was loaded on carbon film grids with 2 μm holes (C-flat, Electron Microscopy Sciences), blotted in an environmentally controlled chamber at 80% humidity within a minute, and vitrified by plunging into liquid ethane at −180 °C (EM GP2 Automatic Plunge Freezer, Leica). The grids with different ice thickness formed at varying freezing conditions were screened using the TF20 TEM under cryogenic conditions to identify the conditions that avoided possible damages (e.g., protein denaturation and complex disassembly) that could be induced by the presence of an air–water interface during vitrification.^59^ A set of 18 cryo-grids was inspected using the TF20 TEM under cryo conditions (−180 °C), ten of which had well-distributed viruses at various orientations and optimal ice thickness facilitating proper imaging. To acquire images for SPA, 300 cryo-movies of 3,000 phages total were recorded using a Falcon 3 camera on the Glacios TEM using Smart EPU software (Thermo Scientific) at defocusing ranging from −2.5 to −3.5 μm. Lastly, to generate a capsid density map from SPA, movies were aligned to obtain cryo-micrographs using the software package BSOFT.^60^ The capsid density map had a resolution estimated to be 17 Å at a Fourier-shell correlation cutoff of 0.3. For image file extraction, UCSF Chimera was used.^61^

For electrocoagulated phages, cryo-EM imaging and follow-up three-dimensional cryo-ET were conducted. Samples of electrocoagulated P1 were scrutinized to ensure a sufficient population of damaged phage particles. Cryo-grids of the screened sample were prepared by following gold nanoparticle addition, blotting, and vitrification. Eight optimal grids were imaged in a high resolution using a Glacios cryo-TEM equipped with an autoloader and Falcon III direct electron detector used for recording cryo-tilt series (in ThermoFisher proprietary electron-event representation (EER) image file format) extended from −60° to +60° stage tilt at incremental steps of 2° with a pixel size of 1.98 Å, a dose of 1.5 e^–^/(Å^2^ tilt), and exposure time of 0.626 s/tilt, resulting in a total dose of 91.5 e^–^/Å^2^ in total. A total of five tilt series with 18 damaged virions were collected. Reconstruction data were collected using ThermoFisher data acquisition software (EPU) followed by an initial alignment of the tomography image series by Inspect 3D software (ThermoFisher Scientific). The final tomography reconstruction was carried out using EMAN2.^62^ For image file extraction, UCSF Chimera was used.^61^

## 3 Results and Discussion

### 3.1 Facile Removal/Inactivation of P1 by Electrocoagulation and Conventional Coagulation

Figure 1A depicts results from a negative control experiment wherein electrodes were dipped in the reactor and removed after 34.6 s (duration needed to electrodissolve the targeted 0.2 mg/L iron concentration) without turning on the power. The contents were then slow-mixed (flocculated) for a total duration of 1 h. Phage concentrations were nearly unchanged during the entire duration of this control experiment, remaining at approximately the initial spiked concentration denoting negligible removal, inactivation, aggregation, or deposition on any of the reactor components.^63,64^ Therefore, all measured transient changes in P1 concentrations were attributed to external FeCl_3_ dosing or *in situ* iron electrodissolution. It is also noted that loss of infective phages during measurements (i.e., syringe-filtration and Na_2_SO_3_ addition) was insignificant (SI Section S4).

**Figure 1:**
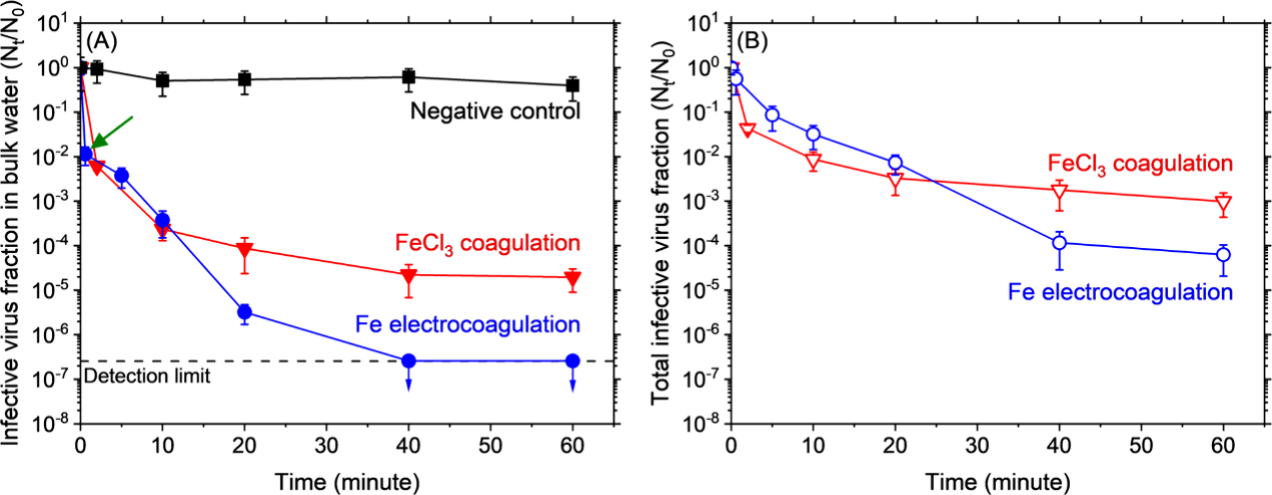
Infective virus fraction temporal profiles in bulk water alone (left panel, A) and the whole suspension (bulk water + flocs, right panel, B). Time-variant concentrations (*N*_t_) were normalized by the initial concentration (*N*_0_). Electrocoagulation results are shown in blue, conventional coagulation in red, and a negative control in black. Symbols represent averages and error bars represent one standard deviation based on duplicated experiments. The green arrow in (A) depicts the time when electrolysis was terminated (34.6 s).

The LRV reached ∼4-logs in 20 min following FeCl_3_ addition (Figure 1A), incrementally approaching ∼5-logs in the next 40 min. Hence, enmeshment and sweep coagulation even with a Fe­(III) dose of only 0.2 mg/L removed/inactivated this tailed phage to the United States Environmental Protection Agency’s primary drinking water standard (LRV = 4) within the typical design residence time for flocculation during conventional treatment.^65^

Electrocoagulation outperformed FeCl_3_ coagulation achieving ∼5.5 LRV in 20 min and surpassed the detection limit (∼7-logs) sometime between 20 and 40 min (Figure 1A). Unlike FeCl_3_, electrocoagulation continuously reduced infective virus concentrations and did not reach an asymptote (excluding the detection limit), demonstrating the advantage of incorporating longer flocculation times in its case. P1 behavior during iron electrocoagulation resembles our recent results with nontailed viral surrogates and was attributed to two mechanisms operating in tandem: i.e., enmeshment by sweep coagulation and inactivation by electroFenton reactions.^14,23,31,52^ P1 LRVs via iron (electro)­coagulation measured herein were much higher than previously reported for other nontailed and short-tailed viruses (MS2, fr, ϕX174, ϕ6, and P22; SI Table S1).^14,31,66^

Since monitoring loss of infective phages from the bulk water alone cannot distinguish between removal and inactivation, evidence for P1 inactivation was pursued by attempting to recover infective phages from suspension by adding beef extract at alkaline pH.^14,39,54^ Therefore, Figure 1B represents total infectivity loss induced by reactive oxygen species (ROS) and/or iron flocs.^14,23,52^ In all samples from the FeCl_3_ experiment (Figure 1B), P1 was only partially recovered using the plaque assay, demonstrating its inactivation simply by enmeshment like ϕ6,^23^ but unlike MS2.^52^ The transient profile of infective viruses thus recovered during FeCl_3_ coagulation mirrored that of the bulk water (Figure 1A) wherein a steep decline was measured in the first 10 min before slowly and only slightly (i.e., ≲1-log) improving over the next 50 min. In the case of electrocoagulation (Figure 1B), substantial inactivation was evidenced by the strong monotonic decline in infective viruses during the first 40 min reaching ≳4-logs. Hence, not only did electrocoagulation better inactivate P1 compared with FeCl_3_ (i.e., by ∼1-log at 60 min) (α = 0.05, *n* = 4), but it did so over longer times needing ≳20 min to outperform conventional coagulation. Again, this emphasizes the advantage of longer flocculation times and the existence of an additional inactivation mechanism during iron electrocoagulation (i.e., electroFenton reactions initiated by anodic and cathodic generation of Fe­(II) and H_2_O_2_, respectively^14,23,31,52^ ). Hence, electrocoagulation inactivated P1 by a combination of “physical” and “chemical” means following contact with iron flocs and reactions with ROS^14,23,52^ (see also Sections 3.3 and 3.4).

It is emphasized that these extremely “favorable” results correspond to a miniscule iron concentration of 0.2 mg/L, which is ∼1 order of magnitude lower than those commonly employed for conventional treatment.^67^

### 3.2 Structure of Intact P1 Virions

Untreated virions were first imaged as a baseline control to identify electrocoagulation-induced structural damages. Cryo-EMs clearly depicted icosahedral heads, long contractile tails, baseplate (white arrows), and tail fibers (Figure 2A), characteristic of the myophage morphology.^68^ Interestingly, a single hollow capsid lacking its genome was present (labeled “Empty capsid”). Its gray value profile computationally measured along the dotted line revealed two intense peaks at the capsid boundaries distinctly demarcating themselves from the interior void (arrowheads in the Figure 2D top panel). All other capsids encapsulated the genome and did not clearly differentiate themselves from their own interiors, an example of which is labeled “Mature capsid” in Figure 2A. Its cross-sectional gray values following the central dotted line (Figure 2D middle panel) showed no obvious distinguishing features at both capsid boundaries at the same locations as the empty capsid (pointed out by arrowheads), but they generally resembled numerous other peaks. Such an indistinct gray scale value distribution could be caused by the internal mass of its genome.^69^ Hence, one of the mature capsids (boxed at Figure 2A bottom) was visualized at higher magnification (Figure 2A inset), which revealed periodic features with ∼2 nm thickness at 2.6 nm internal spacing on average (Figure 2D bottom panel). This strongly agrees with the presence of dsDNA helices concentrically coiled inside the capsid at a uniform interaxial spacing^69–71^ potentially validating the assertion that the genome resulted in the largely indistinguishable profile across the entire capsid and thereby making it difficult to clearly discern its boundaries via image analysis.

**Figure 2:**
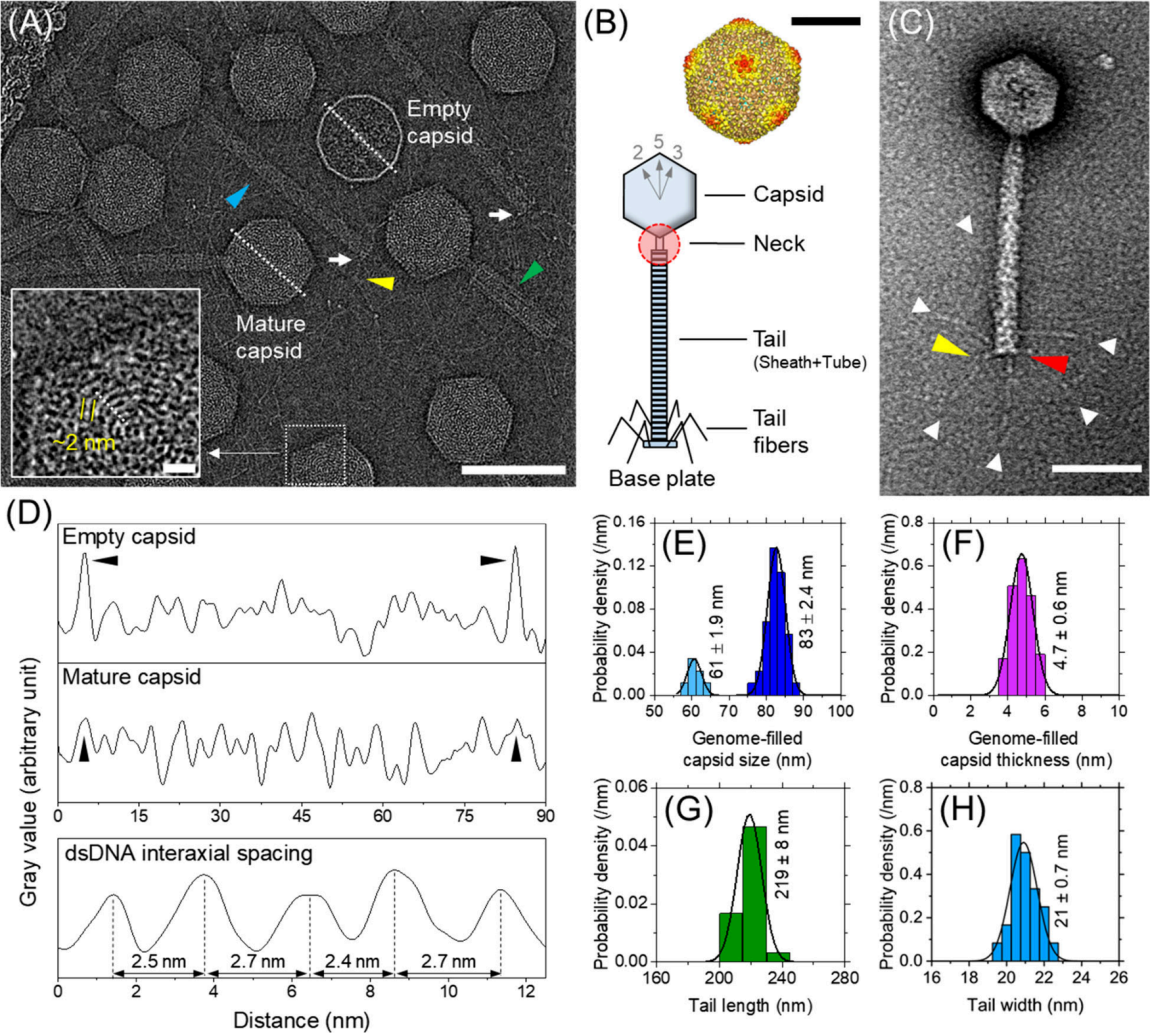
Visualization and structural information on intact P1 prior to coagulation. (A) Cryo-EM of untreated virions (scale bar: 100 nm) showing mature genome-filled capsids and a single empty capsid lacking its genome. The inset (scale bar: 10 nm) is a magnified image highlighting the dsDNA packing for the boxed-out portion of the capsid in the bottom of the larger image. Uncontracted and contracted tails were also visible and marked with cyan and green arrowheads, respectively. The kink in tail fibers is designated with a yellow arrowhead. Baseplates are pointed by white arrows. (B) Top: isosurface representation of the capsid electron density map with icosahedral symmetry (scale: 50 nm), Bottom: phage structure illustration with a neck shaded in red. (C) Conventional TEM of a single virion clearly depicting six tail fibers each marked with a white arrowhead, a distinct tail fiber kink that is marked with a yellow arrowhead, and the baseplate that is marked with a red arrowhead (scale bar: 100 nm). (D) Gray value profiles where the top panel shows the values across an empty capsid following the dotted line in (A), the middle panel shows the values across a fully developed (mature) capsid encapsulating dsDNA following the dotted line in (A), and the bottom panel shows the values for dsDNA packed inside the capsid following the dotted line shown in the inset of (A). (E) The capsid size was bimodally distributed, each of which followed a Gaussian distribution with mean values of 83 and 61 nm (*n* = 44). (F–H) The capsid thickness (*n* = 95), uncontracted tail length (*n* = 20), and uncontracted tail width (*n* = 24), respectively. The average and standard deviation are shown in each panel. The number of measurements (*n*) was based on 106 phage particles in two TEM images and one cryo-EM image. Note that not all particles were suitable for all measurements due to overlap in the images.

Viral structural information was obtained by digitally analyzing EMs, which revealed a bimodal capsid size distribution with the majority (84%) being 83 ± 2.4 nm coexisting with a subpopulation (16%) with smaller capsids (61 ± 1.9 nm) as shown in Figure 2E.^72^ The capsid thickness was measured as 4.7 ± 0.6 nm (Figure 2F). The tail composed of a sheath and an inner tube attached to a vertex whose uncontracted (marked with a cyan color arrowhead in Figure 2A) length and width were 219 ± 8 nm (Figure 2G) and 21 ± 0.7 nm (Figure 2H), respectively.^50,73^ We also observed contracted tails (marked with a green arrowhead in Figure 2A). Even though cryo-EM visualized genomic material (Figures 2A and 2D), demonstrating its superiority over conventional EM, its poor signal-to-noise ratio made P1’s long and thin tail fibers less apparent.^74,75^ Therefore, images were also taken after negative staining (Figure 2C), which clearly showed all viral components, including all six tail fibers (white arrowheads) and the baseplate (red arrowhead), demonstrating the complementary value of conventional TEM to cryo-EM. The kink in tail fibers was visible in both cryo- and conventional TEM images and marked with yellow arrowheads in Figures 2A and 2C, respectively. Cryo-TEM images of intact P1 capsids were used to reconstruct their high-resolution electron density map (Figure 2B top) using the procedure outlined in section 2.4, and structural information obtained from EMs was used to generate its schematic (Figure 2B bottom).

### 3.3 Structure of P1 Virions Damaged by Electrocoagulation

Figure 3A is a cryo-EM image of electrocoagulated P1 phages depicting four capsids suffering local damage (white arrows) and missing their tails (labeled as “a,” “b,” “c,” and “d”) along with four detached tails around them (each marked with an asterisk “*”). Based on their gray-value profiles, all these capsids likely (partially) lacked genomes (SI Section S6). Cryo-EMs at different angles revealed that capsids “a” and “c” were structurally distorted. Therefore, they were boxed out and rotated to various extents and are shown in the top rows of Figures 3B1 and 3B2, respectively. For both these particles, this procedure revealed extensive differences in capsid boundaries compared with intact (untreated) ones based on the rotation angle indicating their heads were no longer well-defined icosahedra and structurally modified. Multiple localized damages on these capsids are marked with white arrows. For clarity, these images were reconstructed at high resolution, and corresponding capsids are depicted in the bottom rows of Figures 3B1 and 3B2, where black arrows denote the same damage locations as in the top rows.

**Figure 3:**
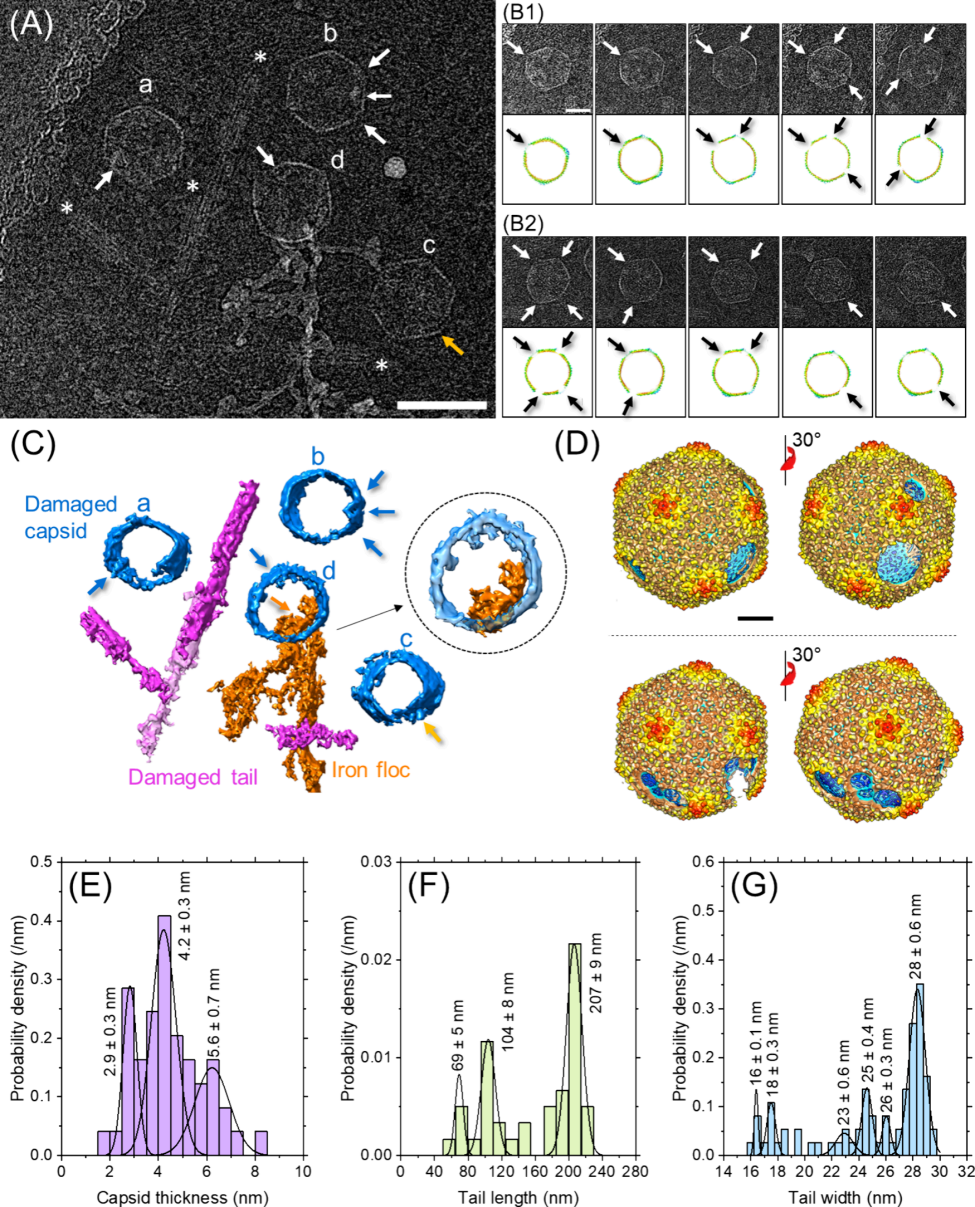
Structural changes of P1 induced by iron electrocoagulation captured by cryo-EM. The sample was taken at the end of electrocoagulation (i.e., *t* = 60 min in Figure 1). (A) Iron electrocoagulated P1 particles (scale: 100 nm). Capsid heads are labeled with alphabets, while tails are marked with asterisks. Capsid damages and discontinuity in capsid are pointed out by white arrows and a yellow arrow, respectively. (B) Distorted capsid layer of ‘a’ and ‘c’ (top row of B1 and B2, respectively) and corresponding high-resolution reconstructions depicting localized damages (bottom rows). (C) Tomography of the scene shown in A. Capsid damage, iron floc penetrating the capsid, and discontinuity on capsid are pointed by blue, orange, and yellow arrow(s), respectively. The inset with adjusted transparency highlights an iron floc penetrating the capsid. (D) Three dimensional electron density maps of P1 particles shown in (B1) (top) and (B2) (bottom) at different angles depicting multiple localized damages on capsid (scale: 20 nm). Structural component dimensions after electrocoagulation; (E) Capsid thickness (*n* = 49), (F) Tail length (*n* = 40), and (G) Tail width (*n* = 74). The number of measurements (n) was based on 155 particles in 5 TEM and 2 cryo-EM images. Note that not all particles were suitable for all measurements due to overlap in the images.

Isosurface representations of density maps of damaged capsids “a” and “c” from Figure 3A are shown in Figure 3D to complement the sliced images in Figures 3B1 and 3B2. Damages were computationally generated on the electron density map of untreated P1 capsid (obtained via SPA, Figure 2B top) to mimic the lost regions of the capsids seen in the cryogenic images of the tilt series.^23^ Because none of these capsids retained their genome, it appears that the extent of damage was sufficient for dsDNA to escape out of its protective head into the harsh external aqueous environment (see also Figures 4B2, 4B3, 4B4, 5D2, and 5E2). Additionally, inspection of several viruses from multiple images suggested that the capsid-tail connection (i.e., neck) was structurally the weakest point since the majority of capsids were found to have lost their tail sections.

**Figure 4:**
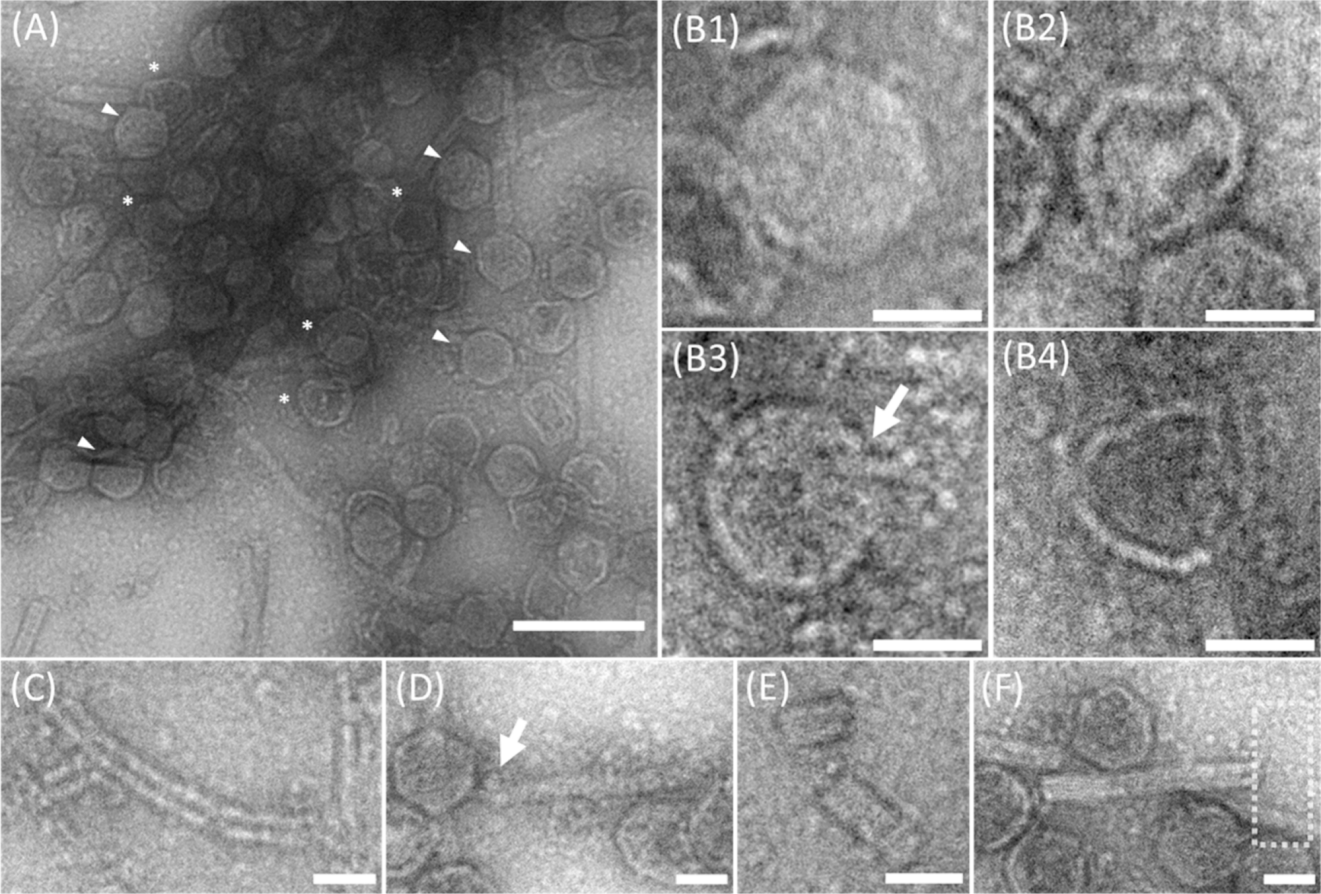
Structural changes in P1 induced by electrocoagulation at the end of 60 min probed by conventional TEM. (A) P1 aggregation by iron flocs (darker area), damaged capsid heads denoted by asterisks, and relatively well-maintained capsid heads marked with arrowheads (scale bar: 200 nm). (B1–B4) Progression and consequence of capsid damage hinted from deformed capsid heads with different features: nearly intact (B1), irregular but closed boundary (B2), dissociation of internal components from an opening (B3), and complete hollow capsid with a wide opening (B4). All scale bars equal 50 nm. (C) A tail missing a capsid head. (D) A kinked head–tail connection (white arrow). (E) Transverse tail fragmentation. (F) Tail fibers detachment (fibers missing in the boxed area). Scale bars in (C–F) equal 50 nm.

**Figure 5:**
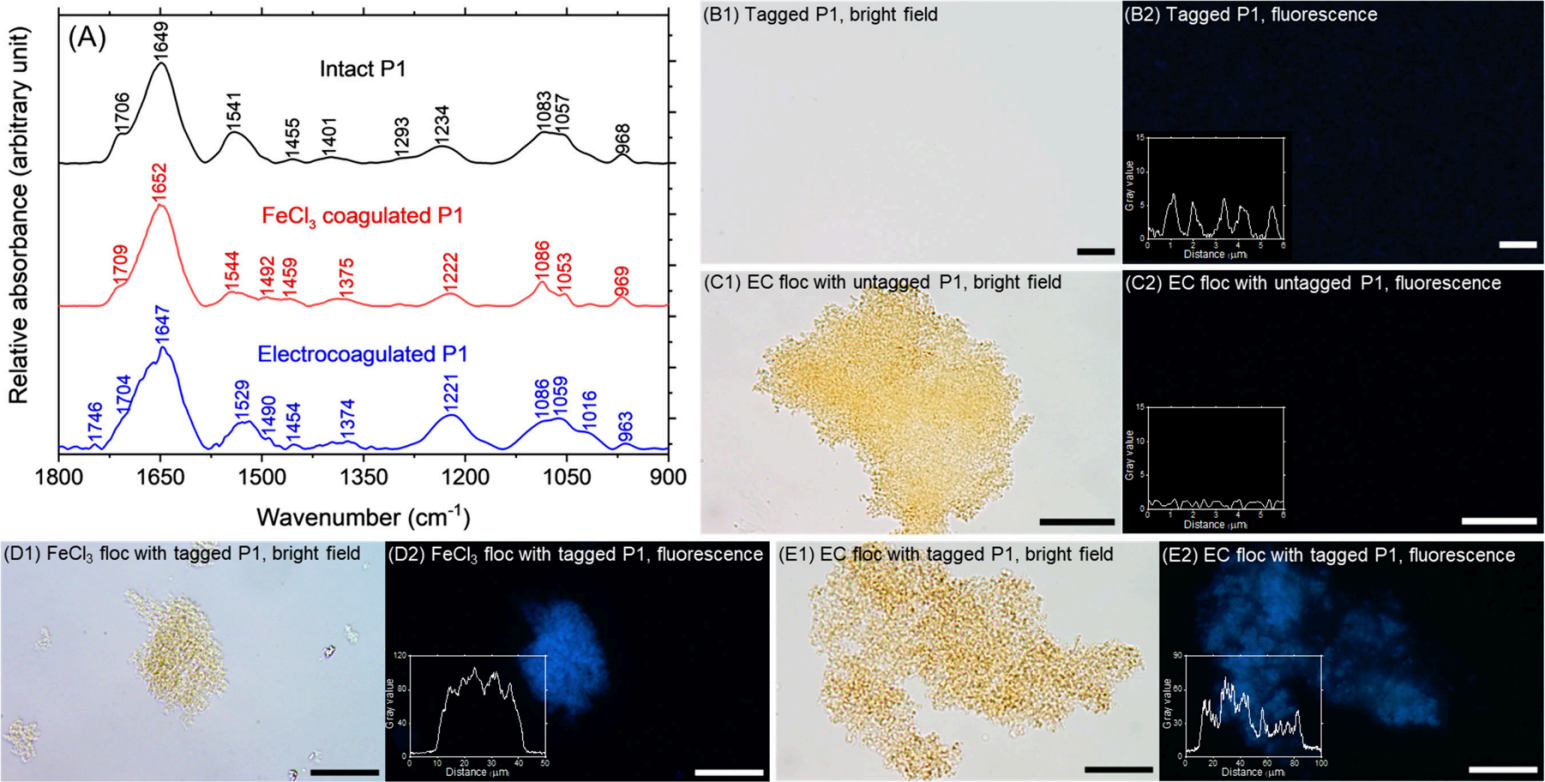
(A) FTIR spectra of intact P1, after conventional FeCl_3_ coagulation and after electrocoagulation. Both coagulated P1 samples were collected at the end of coagulation (i.e., *t* = 60 min in Figure 1). (B1) and (B2) Bright field image of P1 tagged with DAPI and its corresponding fluorescence image, respectively (scale bars: 5 μm). Gray value profile with a periodic pattern in (B2) emphasizes the fluorescence signals. (C1) and (C2) Bright field image and its fluorescence image of iron flocs generated by electrocoagulation, respectively, with untagged P1 as a negative control (scale bars: 10 μm). (D1) and (E1) Bright field images of representative iron flocs after conventional coagulation and electrocoagulation, respectively, (D2) and (E2) Fluorescence images of the same flocs (scale bars: 25 μm). All fluorescence images were acquired under the same conditions: excitation at 340–390 nm, emission at 420 nm, after 300 ms exposure.

Figure 3C is the three-dimensionally regenerated tomogram of the entire scene in Figure 3A generated by using an array of images taken at 61 separate angles. Tails are shown in magenta color confirming the presence of four splintered ones including two that overlapped (the bottom one is represented in faded color). Also, all four capsids imaged in Figure 3A and shown in blue in Figure 3C were missing most of the internal genomic materials. Note that front and back portions of the capsids in (C) were intentionally cut out computationally to expose their internal voids for visual purposes. The cryo-EM of capsid “b” captured dents (Figure 3A white arrows), showing that it was structurally deformed (also pointed by the blue arrows near capsid “b” in Figure 3C). The capsid “c” also showed a discontinuity (yellow arrow) demonstrating its damage. Significant morphological damage to capsid “a” was also observed and marked with a white arrow in Figure 3A. The corresponding locations are also depicted by blue arrows in Figure 3C.

Comparison of tail sections imaged in Figure 3 and Figure 2 revealed that electrocoagulation compressed/contracted along its length, thereby extending the outer sheath diameter from ∼ 21 to ∼ 28 nm. Further, grayscale profiles measured at different regions of the contracted sections did not show strong periodicity along the width (SI Figure S4) indicating that they had lost sheath subunits, thereby exposing the inner tube. Compromised helical periodicity of the contracted tail sheath structure was confirmed by Fourier-transform analysis^76^ computed on the damaged contracted tail sheath where we did not find any reflection that would have indicated periodicity in the compressed sheets (SI Figure S4). Meanwhile, the width of the inner tail tube was measured to be ∼ 9 nm, suggesting that it could withstand the harsh environment induced by electrocoagulation. TEMs of electrocoagulated P1 virions showed several severed tails that had lost the structural rigidity as they appeared to be wavy/flexible, not straight/rigid, as should be the case for P1 (Figure 4). Additionally, the baseplate, which is the nucleating site for the entire tail assembly^77^ was missing in most cases after electrocoagulation, denoting the loss of the virus’ ability to infect its host.^78^ These morphological modifications to electrocoagulated particles provided visual clues to the various damage modes, leading to infectivity loss. Herein, we emphasize qualitative changes to the phage structure upon treatment as documented by cryo-EM, because morphological damage to P1 is expected to depend on coagulation conditions (e.g., pH and dosage) and the presence of organic matter, which were not accounted for in this study.

Next, we focused on a specific capsid in the center of Figure 3A (marked “d”) seemingly in direct contact with chains of spherical/oval-shaped iron precipitates. This network of nanosized products^79^ is consistent with the microstructure of precipitates described in a previous study.^80^ The tomography of the entire scene confirmed this contact and penetration of these chains into the capsid (orange arrow in Figure 3C middle). This is the first direct cryo-EM visualization of flocs penetrating the capsid, causing damage and allowing genome to escape. This indicated that precipitated iron effectively damaged the viral capsid and perturbed internal genomic material possibly in conjunction with ROS (in addition to enmeshing and sweep coagulating P1). Further, the lesion may lead to an unregulated DNA leakage due to abrupt release of the internal pressure originally borne by the intact capsid, which has been shown to accompany integrity collapse.^81,82^

A quantitative analysis was also conducted on P1 structural components after electrocoagulation. Capsid thickness exhibited three peaks in its distribution after treatment (Figure 3E), characterized by a decimated one (2.9 ± 0.3 nm), an enlarged one (5.6 ± 0.7 nm), and one close to the original value (4.2 ± 0.3 nm). When compared to the original monodisperse distribution centered at 4.7 ± 0.6 nm (Figure 2F), this indicates a statistically significant alterations to associated protein (two-sample Kolmogorov–Smirnov test at α=0.05). These thickness changes are symptomatic of capsids’ structural integrity loss and damage, causing genomic material release consistent with missing dsDNA both of which have been described earlier in this section using Figures 3A and 3C (and Figures 4B3, 4B4, 5D2, and 5E2). Fragmented tails (quantified in Figure 3F) demonstrated that tail sheaths and tubes could also be concomitantly damaged and truncated into smaller pieces. Multiple populations of tail widths evolved after treatment, some that were thinner and others that were thicker (Figure 3G) than the original value. Hence, inactivation was achieved by nonselective interactions of electrocoagulation products (i.e., iron precipitates and electroFenton intermediates) with all P1 components.

Electrocoagulation severely aggregated phages and revealed a plethora of morphological alterations under a conventional TEM as shown in Figure 4. Several P1 particles were embedded in iron flocs (dark electron-rich patches) in Figure 4A portraying destabilization by enmeshment like other viruses.^14,23^ Many capsids were deformed by aggregation (depicted by asterisks), corroborating cryo-EM results of morphological damages. Few others were seemingly intact (depicted by arrowheads in Figure 4B1) and maintained their icosahedral shape while retaining their internal mass. Numerous capsids were severely damaged having highly irregular outlines, but their boundaries were continuous, thereby preserving their genome, one example of which is shown in Figure 4B2. Other capsids had discontinuous boundaries potentially allowing genomic materials to escape (Figure 4B3).^81^ Some capsids were completely ruptured and empty with a negatively stained internal volume (Figure 4B4). Hence, both conventional TEM and cryo-EM indicated DNA leakage after coagulation, which is further discussed in the following section.

Tails were also altered, demonstrating nonselective attack by iron electrocoagulation. Completely disconnected tails (Figure 4C and Figures 3A and 3C) indicated electrocoagulation’s ability to sever its connection to the capsid, which can also be inferred from a kinked connection between them (pointed by the arrow in Figure 4D).^83,84^ Fragmented tails (Figure 4E) demonstrated concomitant damage to sheaths and tubes. Further, fibers and baseplates were also detached from tails, one example of which is shown in Figure 4F as a dotted rectangle. Indiscriminate damages to receptor-binding proteins indicates inactivation because it has been reported that at least three tail fibers need to interact with receptors for genome injection into the host.^85^ Therefore, in addition to sweep coagulating P1, electrocoagulation inactivated it by inducing major structural damages to its capsid, tail, tail fibers, and baseplate, as well as allowing DNA to escape thereby disallowing the phage’s ability to infect the host.^68^ Because P1 was not imaged after FeCl_3_ addition in this study, its morphological alterations (leading to inactivation) via conventional iron coagulation strictly remains unknown, although its removal can be strongly attributed to enmeshment and sweep flocculation because of iron precipitation.^14^

### 3.4 Alterations to Phage Biochemistry via Electrocoagulation and Conventional Coagulation

FTIR spectra of intact and (electro)­coagulated phages in the fingerprint region (1800–900 cm^–1^) are compared in Figure 5A to obtain clues to potential conformational and compositional changes to proteins (not to quantify protein oxidation) and nucleic acids brought about by treatment.^73,86^ Sample preparation, peak assignments, and corresponding contributors are detailed in SI Section S8.

Untreated P1 yielded several peaks associated with its known proteinaceous components (capsid, tail, and tail fibers) and genomic material (dsDNA) obtained from the literature.^50,73,77^ For example, those centered at 1649, 1541, and 1293 cm^–1^ were identified as characteristic amide I, II, and III vibration bands of the polypeptide backbone of proteins, respectively.^87^ Peaks at 1455 and 1401 cm^–1^ were assigned to δ_as_(CH_3_) and δ_s_(CH_3_), respectively, of protein side chains containing methyl groups (i.e., alanine, valine, isoleucine, leucine, methionine, and threonine) which are found throughout its head, tail sheath, and tail fibers.^77,88^ The peak at 1401 cm^–1^ can also be attributed to ν_s_(COO^–^) of aspartate and glutamate.^77,88^ Peaks associated with DNA backbones (1234, 1083, 1057, and 968 cm^–1^ for ν_as_(PO_2_^–^), ν_s_(PO_2_^–^), ν­(C–O), and C–C, respectively) and bases (1706 cm^–1^ for C=O of thymine and guanine) were also visible.^87^

Spectra after electro- and conventional coagulation noticeably differed from untreated viruses, suggesting that both treatment approaches modified phage proteins consistent with inactivation and microscopic observations. For example, the amide II relative intensity was 12.3% for intact P1, which decreased to 4.9% after adding FeCl_3_ and to 9.0% following electrocoagulation. These changes in δ­(N–H) and ν­(C–N) suggest conformational alterations.^89,90^ The shape of the amide I envelope, which is sensitive to secondary structures of protein backbones^87^ also changed with both types of coagulation indicating structural deformations. The amide I band was deconvoluted to quantify changes in relative amounts of protein secondary structures induced by (electro)­coagulation.^14,91^ One prominent change was for aggregated and unordered structures, which comprised only 17% in untreated P1 but increased significantly to 56% after conventional coagulation and 44% after electrocoagulation (230% and 160% increase, respectively, and shown in SI Figure S5 and Table S4). Importantly, a new carbonyl peak (1746 cm^–1^) emerged exclusively after electrocoagulation (not FeCl_3_ coagulation) symptomatic of protein oxidation,^92^ attributed to electroFenton reactions.^14,52^

Specific chemical interactions between phage proteins and iron flocs were evidenced by a large red shift of the ν_s_(COO^–^) peak from 1401 cm^–1^ (intact P1) to 1375–1374 cm^–1^ after both types of coagulation indicating inner-sphere complexation of virus carboxylate groups with Fe­(III) in flocs like bacteria^93^ and organic moieties^94,95^ or soluble Fe­(II) generated by electrocoagulation.^52,96^

Protein–iron interactions, protein oxidation, and protein secondary structure alterations might rupture/collapse capsids leading to DNA exposure/leakage^81^ as expected from (cryo)­EM in Figures 3 and 4. The possibility of expelled DNA outside the capsid was probed by bright field (Figures 5D1 and 5E1) and corresponding epifluorescence (Figures 5D2 and 5E2) microscopy of flocs generated by electrocoagulation and conventional coagulation, respectively, of DAPI-stained P1 stock (see SI Section S9). All (electro)­coagulated phages exhibited a strong fluorescence signal compared to control samples (Figures 5B2 and 5C2) indicating free-floating dsDNA on flocs consistent with genomic loss visualized by cryo-EM in Figure 3 and room temperature EM in Figure 4. Nucleic acid damage by electrocoagulation and FeCl_3_ coagulation was further evidenced by changes to viral DNA-associated peaks arising from interactions with iron. For example, the ν_as_(PO_2_^–^) band red-shifted from 1234 cm^–1^ for intact P1 to the same wavenumber (1222–1221 cm^–1^) after both coagulation types, suggesting conformational alterations of the DNA backbone^97^ were potentially dominated by interactions with Fe­(III) in flocs.^98^ Further, electrocoagulation nearly doubled the ν­(C–O) to ν_s_(PO_2_^–^) ratio (from 0.93 to 1.74) and more than doubled the ν_as_(PO_2_^–^) to ν_s_(PO_2_^–^) ratio (from 1.2 to 2.7) compared to intact P1. These ratiometric indices indicate that electrocoagulation fragmented viral dsDNA potentially due to electroFenton-induced oxidative stress.^99^ In contrast, conventional coagulation significantly reduced these indices; ν­(C–O) to ν_s_(PO_2_^–^) ratio reaching 0.2 and ν_as_(PO_2_^–^) to ν_s_(PO_2_^–^) ratio becoming 0.7, potentially signifying different (nonoxidative) dominant mechanisms of DNA damage.

## 4 Implications

Extremely facile removal/inactivation of P1 by FeCl_3_ suggests that other phages with the myovirus morphology will also be generally well-controlled when iron is employed to (electro)­coagulate municipal wastewater and sludge.^100^ High P1 LRVs by iron electrocoagulation and conventional coagulation even at a low coagulant dose contrasts the relatively refractory behavior of itself and other tailed phages during ozonation (e.g., T4),^37^ advanced oxidation, zerovalent iron, or electrochemical treatment (e.g., T4 and T7),^38,40,41^ and coagulation with (poly)aluminum salts (e.g., P1, T4)^39^ (SI Table S2). Substantial variances in the fate of tailed viruses in different treatment processes and large disparities in the fate of dissimilar viruses in a given treatment process point to the need to quantify LRVs of individual unit operations using disparate viruses especially in light of the multiple barrier approach^9,42,43^ to ensure public health safety. This is particularly relevant because novel zoonotic viruses can be anticipated to emerge, spread, and find their way into our water supplies due to their evolution, climate change, and/or human activity.^6,101–103^ Additionally, both iron conventional coagulation and electrocoagulation conform to California’s DPR requirement of implementing technologies with “diverse treatment mechanisms”^11^ (called “orthogonal” in the pharmaceutical industry^104^) because they mitigate microorganisms using principles disparate from membrane filtration, chemical disinfection, and advanced oxidation.

Easy control of a tailed phage with a nonenveloped capsid shown herein and of a nontailed phage with an enveloped capsid by iron coagulation and electrocoagulation^14,23^ indicates that nontailed phages with nonenveloped capsids might be better suited to serve as conservative surrogates for both of these unit processes. These observations can be generalized to infer that nontailed phages would be better surrogates than tailed phages to evaluate viral LRVs during water/wastewater/secondary effluent coagulation.^42,105–107^ One possibility is the MS2 phage, which has been recommended by environmental virologists^44^ given its small size, stability imparted by its proteinaceous capsid (i.e., nonenveloped), lack of a tail,^3^ and availability of extensive data sets allowing interlaboratory and interprocess comparisons.^29,30,64^ Further, high LRVs of the tailed virus surrogate P1 achieved by electrocoagulation (with previous work targeting nontailed or short-tailed phages^31,66,108^ ) adds to our body of knowledge suggesting the suitability of this technology for small-scale distributed water/wastewater treatment. Importantly, experiments reported herein were conducted in a well-controlled environment using synthetic water, which may result in different viral adhesion, aggregation, and inactivation behaviors compared to real-world conditions. When discussing implications for regulatory LRV credits and microbial risk assessments, it is imperative to evaluate these potential variations and consider how laboratory findings can be extrapolated to an actual wastewater matrix. Finally, we emphasize that cryo-EM generates complementary information (morphology and structure) to techniques such as polymerase chain reaction^23,30,105^ (for genomic content) and mass spectrometry^105,109^ (for proteins) that are more commonly employed in environmental science and engineering to study damages imparted to viruses following treatment. As such, characterizing viruses with a wide suite of instrumental techniques will provide a comprehensive and mechanistic picture of virus removal/inactivation. In this context, we are currently identifying capsid protein modifications induced by electro- and conventional coagulation using proteomics tools (e.g., matrix-assisted laser desorption ionization time-of-flight mass spectroscopy),^105,109^ which we hope to report soon.

## Supplementary Material



## Notes

Details of phage stock preparation, summary of experimental designs, flowchart of EM analysis, control experiments for phage assay, literature summaries, gray-value profiles of damaged capsids, Fourier-transform estimation of tail periodicity, details of IR analysis (sample preparation, peak identification/assignment, peak decomposition), and details of phage tagging protocols (PDF)
