# Supplementary material for: Facile Control of a Tailed Virus Surrogate by Iron Conventional Coagulation and Electrocoagulation
Source: Environ Sci Technol. 2025 Jul 30;59(31):16697–708. doi: 10.1021/acs.est.5c02792 (PMC12355955; doi:10.1021/acs.est.5c02792)
Supplement: Supplementary file 1 [file es5c02792_si_001.pdf]

SUPPORTING INFORMATION

Facile Control of a Tailed Virus Surrogate by Iron Conventional Coagulation and Electrocoagulation

Kyungho Kim[1], Anindito Sen[2], Shankararaman Chellam[1,3,*]

[1]Department of Civil & Environmental Engineering, Texas A&M University, College Station, TX 77843-3136

[2]Microscopy and Imaging Center, Texas A&M University, College Station, TX 77843-2257

[3]Department of Chemical Engineering, Texas A&M University, College Station, TX 77843-3122

Corresponding author: S. Chellam, Zachry Department of Civil & Environmental Engineering, Texas A&M University, College Station, TX 77843. Phone: (979) 458 5914; chellam@tamu.edu

Number of pages: 15
Number of figures: 5
Number of tables: 4

Section S1. Virus Propagation and Purification Protocol. *Escherichia coli* was grown in Luria-Bertani (LB) broth (10 g/L tryptone, 5 g/L yeast extract, 10 g/L NaCl, and 10 mM $MgSO_4$) until its optical density at 600 nm reached 0.35, which was then inoculated with P1 at multiplicity of infection of ~1 in presence of 5 mM $CaCl_2$. A 100 µL mixture was added to 10 mL soft LB agar (LB broth amended with 5 g/L agar and 5 mM $CaCl_2$) and poured onto a plate (LB broth amended with 15 g/L agar and 5 mM $CaCl_2$) and incubated at 37 °C until a clear phage lawn appeared because of confluent lysis. The lawn was collected and centrifuged (10,000 g, 10 min, 4 °C) after which supernatant was recovered, purified using a 0.45 µm polyethersulfone filter, pelletized (15,000 g, 24 hours, 4 °C), and resuspended in 3 mL TM buffer (50 mM Tris-HCl, 100 mM NaCl, 8 mM $MgSO_4$, pH 7.3) overnight at 4 °C. Afterwards, the resultant was passed through CsCl layers (1.3~1.6 g/cm^3 prepared in SM buffer, 104,000 g, 24 hours, 4 °C). The phage band was recovered, pelletized (15,000 g, 24 hours, 4 °C), and resuspended in 1 mL TM buffer overnight at 4 °C.

Section S2. Summary of Experimental Sets, Sampling Intervals, Sample Preparation, and Corresponding Analysis.

Experiment	Sampling	Sample Preparation	Analysis
○ Negative control (No coagulant, pH 6.5)	▪ At t= 2, 10, 20, 40, and 60 min	✓ 0.1 mL suspension	➢ Infectivity assay (bulk concentration)
○ $FeCl_3$ coagulation (0.5 mg/L Fe, pH 6.5)	▪ At t= 2, 10, 20, 40, and 60 min	✓ 1 mL suspension → 0.45 µm filtering<br>✓ 0.1 mL suspension → 0.45 µm filtering → added to 0.9 mL 6% beef extract	➢ Infectivity assay (bulk concentration)<br>➢ Infectivity assay (total concentration)
	▪ At t= 60 min	✓ 50 mL suspension → Pelletizing by centrifugation (15000 g, 24 hr, 4 °C)	➢ FTIR
○ Electrocoagulation (0.5 mg/L Fe, pH 6.5)	▪ At t= 2, 10, 20, 40, and 60 min	✓ 1 mL suspension → 0.45 µm filtering + 25 mM Na_2SO_3<br>✓ 0.1 mL suspension → 0.45 µm filtering + 25 mM Na_2SO_3 → added to 0.9 mL 6% beef extract	➢ Infectivity assay (bulk concentration)<br>➢ Infectivity assay (total concentration)
	▪ At t= 60 min	✓ 50 mL suspension → 25 mM Na_2SO_3 → Pelletizing by centrifugation (15000 g, 24 hr, 4 °C)	➢ FTIR
		✓ 50 mL suspension → 25 mM Na_2SO_3 → Pelletizing by centrifugation (15000 g, 24 hr, 4 °C) → Resuspended in 1 mL TM-buffer	➢ Conventional TEM*<br>➢ Cryo-EM & Tomography*

* Further details are shown in Section S3.

Section S3. Flowchart of Electron Microscopic Imaging and Downstream Computational Analysis.

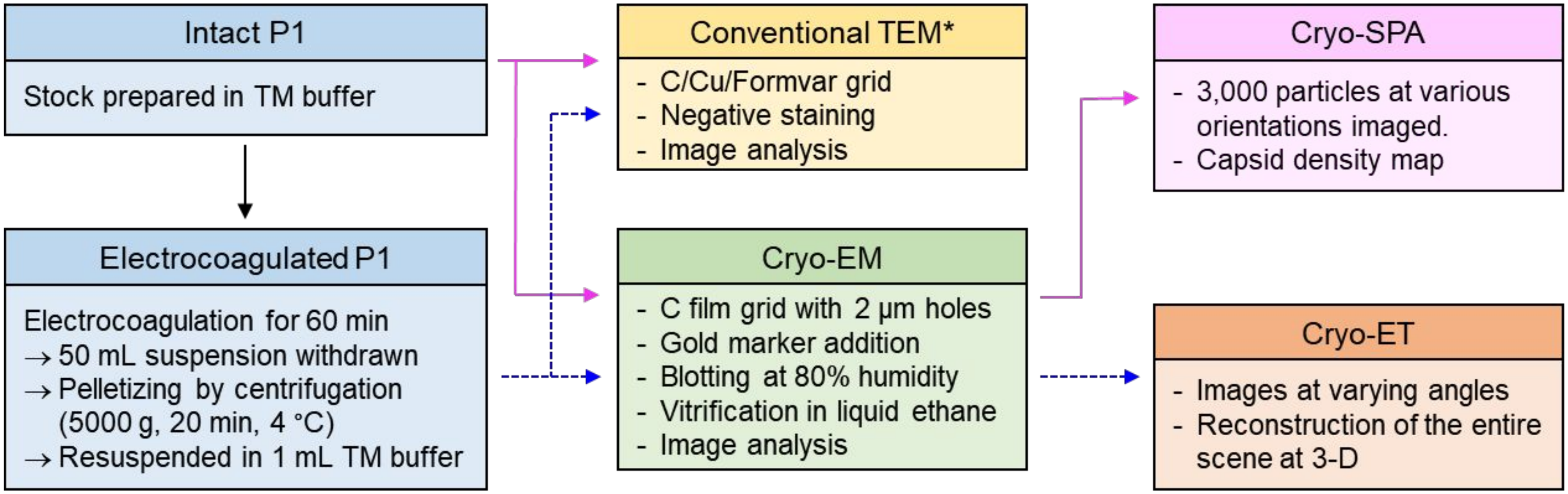

* Conventional TEM was also used to screen samples suitable for cryo-EM.

Section S4. Control Experiments for Infective Virus Concentration Assay. In this study, infective P1 phages remaining in bulk water were assayed after 0.45 μm polyethersulfone (PES) syringe filtration and 25 mM Na_2SO_3 addition. Hence, the effect of these two procedures was quantified prior. A *t*-test for both control experiments at a 95 % confidence interval (OriginPro 2018) indicated the statistically insignificant difference in mean concentrations.

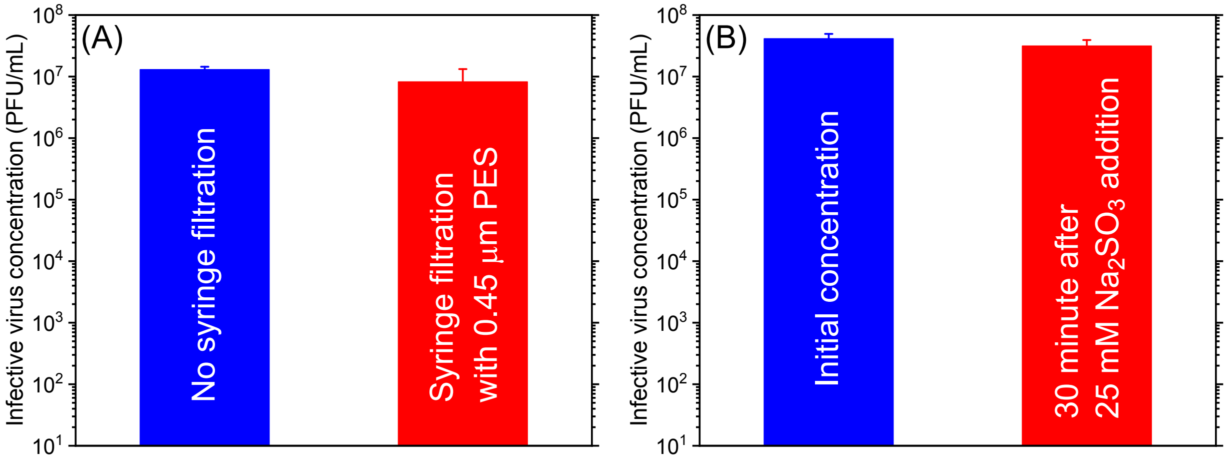

Figure S1. Negligible P1 infectivity reduction by syringe filtration and Na_2SO_3 addition.

Section S5. Summary of Literature Review on Relevant Studies. Results from previous studies on conventional chemical iron coagulation (CC) and iron electrocoagulation (EC) on viruses are summarized in Table S1. Note that studies with organic-containing solutions were excluded. Table S2 summarizes literature where tailed phages demonstrated relatively higher resistance against various processes.

Table S1. Literature review on iron (electro)coagulation of various viruses.

Virus	Structure	Solution	Coagulation	Dose (mg/L as Fe)	Log reduction in bulk water	Ref.
MS2	Non-enveloped<br>Non-tailed<br>Icosahedral capsid<br>ssRNA	$NaHCO_3$ (3 mM)<br>$CaCl_2$ (1 mM)<br>pH 6.4, 8.2	EC	5 ~ 20	0.2 ~ 3.5 at pH 6.4 in 20 minutes<br>0.5 ~ 1.9 at pH 8.2 in 20 minutes<br>* Infectivity assay was performed for the supernatant after centrifugation.	[1]
		$NaNO_3$ (3.3 mM)<br>$NaHCO_3$ (1 mM)<br>pH 6, 7, 8		2.3	2.5 at pH 6<br>3.5 at pH 7<br>5.3 at pH 8	[2*]
ϕX174	Non-enveloped<br>Non-tailed<br>Icosahedral capsid<br>ssDNA	NaCl (175 mM)<br>pH 5.5, 7.5, 8.5	EC	52.1	0.8 ~ 5.2 depending on the flocculation condition	[3]
		$NaNO_3$ (3.3 mM)<br>$NaHCO_3$ (1 mM)<br>pH 6, 7, 8		2.3	0.6 at pH 6<br>2.0 at pH 7<br>1.1 at pH 8	[2*]
fr	Non-enveloped<br>Non-tailed<br>Icosahedral capsid<br>ssDNA	$NaNO_3$ (3.3 mM)<br>$NaHCO_3$ (1 mM)<br>pH 6, 7, 8	EC	2.3	5.5 at pH 6<br>2.7 at pH 7<br>1.1 at pH 8	[2*]
			CC	2.3	0.9 at pH 7	
P22	Non-enveloped<br>Short-tailed<br>dsDNA		EC	2.3	>5.7 at pH 6<br>2.3 at pH 7<br>2.0 at pH 8	[2*]

* Infectivity assay was performed after syringe filtration with 0.45 μm PTFE filters

Table S2. Summary of studies where tailed phages were directly compared to non-tailed ones.

Process	Experiments and measurements	Notable results	Ref.
Ozonation	○ Viruses were treated at varying ozone (O_3) exposure extent (i.e., O_3 concentration and exposure time).<br>○ Second-order kinetic constants were estimated.	• Inactivation kinetics of tailed T4 was slower by 1.5 and 2.5 times than those of non-tailed MS2 and Qβ, respectively.	4
Aluminum-based coagulation	○ Phages were coagulated with aluminum-based coagulants<br>○ Phages captured by flocs were recovered and quantified.	• Tailed P1 and T4 were recovered more from aluminum flocs than non-tailed MS2 and Qβ in numerous experimental conditions (e.g., 1 mg-Al/L aluminum sulfate).	5
UV/H_2O_2	○ Microbes including phages were treated by UV/H_2O_2.<br>○ Hydroxyl radical (·OH) concentration during UV/H_2O_2 was quantified using *para*-chlorobenzoic acid.<br>○ Log inactivation was estimated at varying exposure extent (i.e., ·OH concentration and exposure time, CT-value).	• CT value for 0.5-log inactivation for tailed T7 (1.47×10^{-13} M·min) was higher than that of MS2 (1.18×10^{-13} M·min).	6
Zero-valent ion nanoparticle (nZVI)	○ Phages were mixed with nZVI for 6 hours.<br>○ Viable phages were quantified every hour.<br>○ Control experiments with ferrous and ferric ions were also conducted.	• nZVI treatment for 6 hours was more effective against non-tailed MS2 (2-log reduction) than tailed T4 and T7 (1.5-log and 0.5-log reduction, respectively).<br>• Tailed T4 was significantly more resistant against Fe(II) (~1.5-log) than non-tailed MS2 (8-log).	7
Iron EC	○ Viruses were treated with Iron EC under various conditions.<br>○ Viable viruses were quantified after filtering the sample with a 0.45 μm PTFE syringe filter.<br>○ Control experiments with ferrous and ferric iron were also conducted.	• Short-tailed P22 was less attenuated by EC (~2.3-log) compared to MS2 (~3.6-log) at pH 7.	2
Electrochemical oxidation/filtration	○ Titanium suboxide doped graphenes with different doping amounts were synthesized in the form of a plate and filter.<br>○ Phages were electrochemically treated in batch mode using the plate electrode and in flow-through mode using the filter under various voltages.	• Overall, non-tailed MS2 was more susceptible than tailed T4 (e.g., >6-log vs. 1-log at 2.5 V in a batch mode after 6 hours using a graphene anode doped with 10% TiO_X).	8

Section S6. Gray-value Profiles of Electrocoagulated P1 Capsids. Gray-value profiles of electrocoagulated P1 capsids were obtained along with the yellow-dotted lines (Figure S2). Unlike the profile of the intact capsid shown in Figure 2D middle panel in the manuscript, these capsids appear to have irregular profiles (Figure S3) indicating a (partial) loss of genomic material inside.

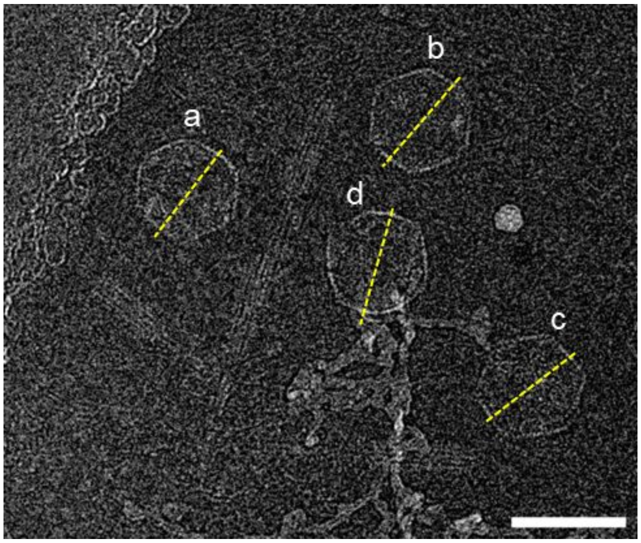

Figure S2. Cryo-EM of electrocoagulated P1 phages where four capsids seemingly missing internal genomes. Gray values were read along the yellow-dotted lines as shown in Figure S3.

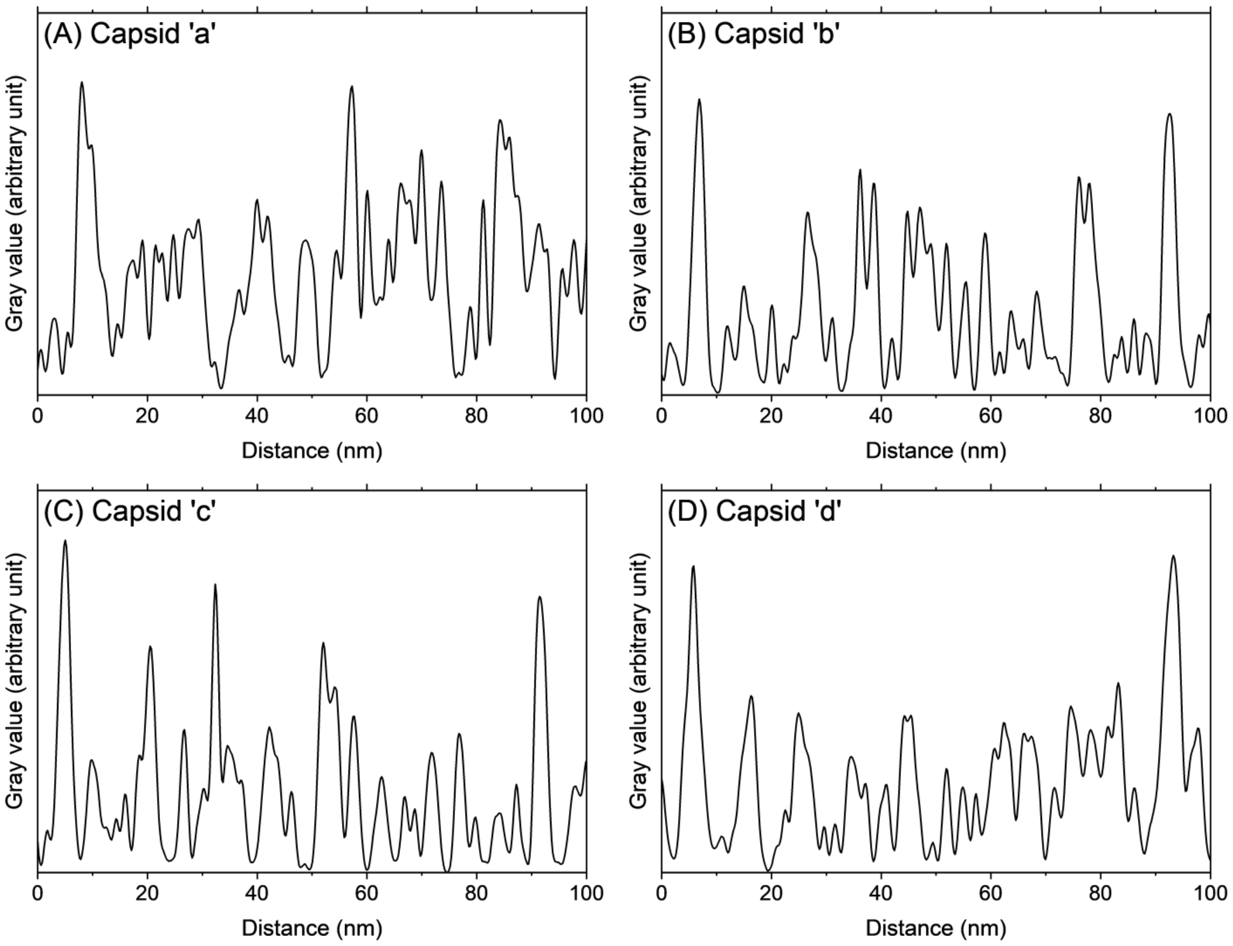

Figure S3. Gray-value profiles of P1 capsids along the yellow-dotted lines shown in Figure S2.

Section S7. Further Analysis on Damaged Tail.

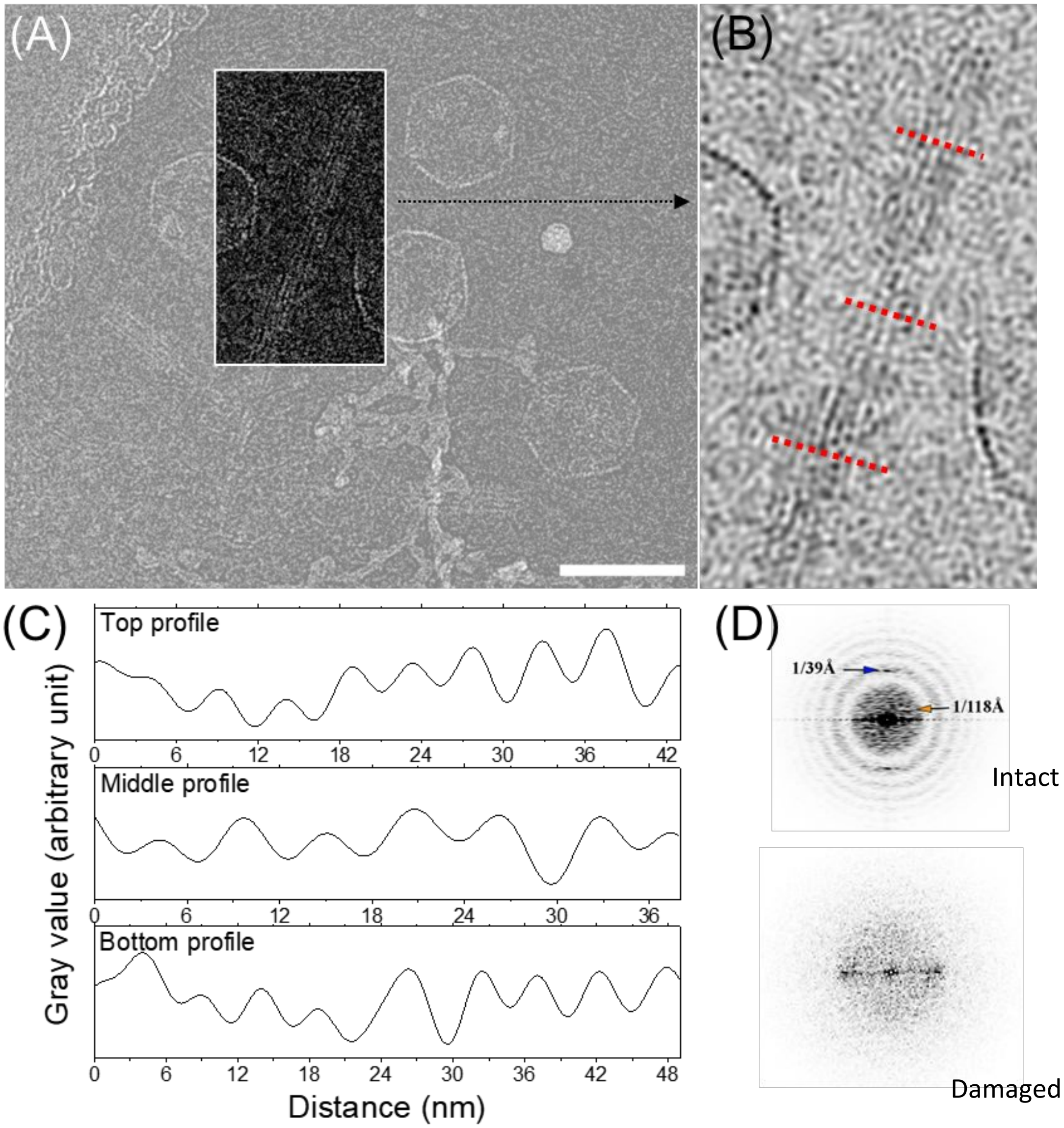

Figure S4. Detailed analysis of the tail damages and the consequent loss of helical periodicity. The damaged tail of interest (A) was magnified and monochromatic-inversed (B). (C) shows the gray value profiles along the three red-dotted lines in (B). The thickness of the middle section of the contracted damaged tail in (B) was much lower than the two ends. (D) Fourier-transform estimation of helical periodicity of intact (top) and electrocoagulated P1 tail (bottom). The axial reflection at 1/39 Å position represents the axial rise of the helix of the phage tube. The reflection at 1/118 Å shows the repeat of the protein subunits forming the tail.

Section S8. ATR-FTIR Sample Preparation and Measurement Protocols. For intact P1 spectrum, phages in a purified stock (~10^{11} PFU/mL) were pelletized by centrifugation (15000 g, 24 hours, 4 °C). For coagulated phage spectrums, the suspension was withdrawn at the end of the coagulation (i.e., t= 60 minutes in Figure 1), treated with 25 mM Na_2SO_3 (for electrocoagulated phages), pelletized by centrifugation (15000 g, 24 hours, 4 °C). The resulting pellets were directly mounted on the iTX accessory of Nicolet iS10 (Thermo Fisher Scientific) equipped with an Ever-Glo MIR source, KBr beam splitter, and DTGS detector. The spectrum was an average of 128 scans recorded at 4 cm-1 resolution, which was ATR- and auto-based line corrected on OMNIC software (Thermo Fisher Scientific). The spectrum was further processed on Origin 2018 (OriginLab) via normalization with respect to the largest peak intensity and Savitzky-Golay smoothening. Spectrum analysis such as peak identification by taking second derivatives and peak decomposition was also performed on Origin 2018.

Table S3. FTIR peak assignments and corresponding contributors.[9-11]

Wavenumber (cm^{-1})	Assignment	Contributor
1746	$\nu(C{=}O)$	Carbonyl groups
1709, 1706, 1704	$\nu(C{=}O)$	Thymine and guanine
1652, 1649, 1647	Amide I, $\nu(C{=}O)$	Protein backbone
1544, 1541, 1529	Amide II, δ(N-H) and ν(C-N)	Protein backbone
1492, 1490	C-H	Guanine
1459, 1455, 1454	$\delta_{as}(CH_3)$	Protein side chains containing methyl groups
1401	$\delta_s(CH_3)$<br>or $\nu_s(COO^-)$	Protein side chains containing methyl groups<br>or aspartate and glutamate
1375, 1374	$\nu_s(COO^-)$	Aspartate and glutamate coordinated with Fe(II)/Fe(III)
1293	Amide III, δ(N-H) and ν(C-N)	Protein backbone
1234	$\nu_{as}(PO_2^-)$	DNA backbone
1222, 1221	$\nu_{as}(PO_2^-)$	DNA backbone coordinated with Fe(II)/Fe(III)
1086, 1083	$\nu_s(PO_2^-)$	DNA backbone
1059, 1057, 1053	ν(C-O)	DNA backbone
1016	-	DNA
969, 968, 963	C-C	DNA backbone

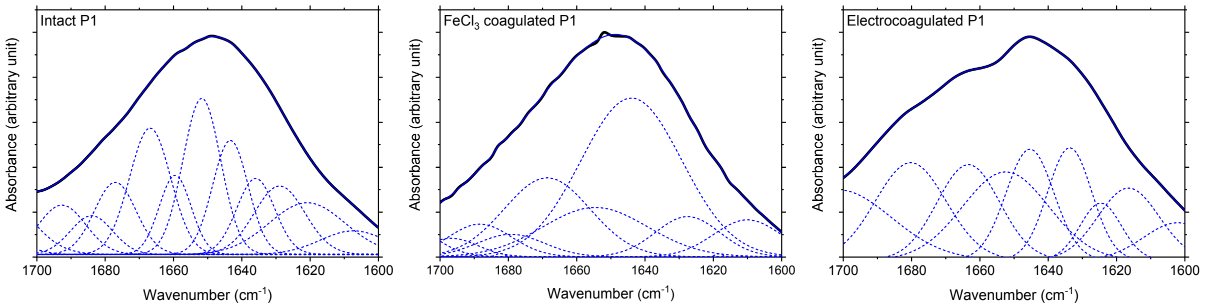

Figure S5. Amide I peak decomposition for quantifying the relative amount of protein secondary structures for intact and coagulated P1 virions. Thick black lines represent original amide I spectra while blue dotted and solid lines are subcomponents and cumulative peaks of them, respectively.

Table S4. Summary of the relative amount of secondary structures based on amide I decomposition.[11-14]

Secondary structure	Intact P1		$FeCl_3$ coagulated P1		Electrocoagulated P1	
	Wavenumber (cm^{-1})	Relative area (%)	Wavenumber (cm^{-1})	Relative area (%)	Wavenumber (cm^{-1})	Relative area (%)
**Aggregated strand**	-	-	1610<br>1618	6.8<br>0.1	1616	11.7
**β-sheet**	1621<br>1629<br>1636	12.1<br>10.6<br>8.8	1628	7.1	1625<br>1634	6.2<br>13.2
**Unordered structure**	1643	12.4	1644	43.7	1645	14.2
**α-helix**	1652	18.1	1655	14.1	1652	20.3
**Turns**	1660<br>1667	7.7<br>16.0	1669	18.5	1663	15.9
**β-sheet**	1677	9.2	1679	4.1	-	-
**Aggregated structure**	1684	5.0	1682<br>1688	0.1<br>5.6	1680	18.3

Section S9. Phage DNA Exposure due to Capsid Breakage. P1 phage DNA was conjugated with 4′,6-diamidino-2-phenylindole (DAPI) as previously described[15] to visualize phage DNA before and after electrocoagulation. A 500 µL of P1 phage stock (~10^{11} PFU/mL) was mixed with an equal volume of DAPI solution (20 µg/mL in deionized water) and incubated for 30 minutes in dark at room temperature. Phages were then rinsed 3 times with SM buffer using a 100 kDa centrifugal filter unit (Amicon Ultra-15, Millipore) at 2,000 g for 5 min to remove excessive DAPI dyes. A control sample was also prepared following an identical protocol but without phages to ensure a negligible contribution of unconjugated DAPI molecules to fluorescent signals. All images were taken with Olympus BX-53. For fluorescence images, samples were excited at UV wavelengths (340-390 nm), and fluorescence signals were captured through a 420 nm long pass filter. It is noted that the exposure time was fixed at 300 milliseconds. No objects were discerned in a bright field mode (Figure 5B1 in the manuscript) whereas dimmed blue dots in approximately 250 nm size appeared in a fluorescence mode (Figure 5B2 in the manuscript). Fluorescence signals were confirmed by a gray value profile where periodic peaks were contrasted from the background (inset of Figure 5B2 in the manuscript). Further, a control sample was prepared and imaged. Electrocoagulation was performed with a blank stock prepared following the identical protocol without the addition of any P1 phages. As indicated by Figures 5C1 and 5C2 in the manuscript, iron flocs were not self-fluorescent, and DAPI dyes were marginally carried over after the rising protocol.

REFERENCES

(1) Kim, K.; Narayanan, J.; Sen, A.; Chellam, S. Virus Removal and Inactivation Mechanisms during Iron Electrocoagulation: Capsid and Genome Damages and Electro-Fenton Reactions. *Environmental Science & Technology* **2021**, *55* (19), 13198-13208. DOI: 10.1021/acs.est.0c04438.

(2) Heffron, J.; McDermid, B.; Maher, E.; McNamara, P. J.; Mayer, B. K. Mechanisms of virus mitigation and suitability of bacteriophages as surrogates in drinking water treatment by iron electrocoagulation. *Water Research* **2019**, *163*, 114877. DOI: 10.1016/j.watres.2019.114877.

(3) Bicudo, B.; van der Werff, B.-J.; Medema, G.; van Halem, D. Disinfection during Iron Electrocoagulation: Differentiating between Inactivation and Floc Entrapment for *Escherichia coli* and Somatic Coliphage ØX174. *ACS ES&T Water* **2022**, *2* (10), 1707-1714. DOI: 10.1021/acsestwater.2c00230.

(4) Wolf, C.; von Gunten, U.; Kohn, T. Kinetics of Inactivation of Waterborne Enteric Viruses by Ozone. *Environmental Science & Technology* **2018**, *52* (4), 2170-2177. DOI: 10.1021/acs.est.7b05111.

(5) Matsui, Y.; Matsushita, T.; Sakuma, S.; Gojo, T.; Mamiya, T.; Suzuoki, H.; Inoue, T. Virus inactivation in aluminum and polyaluminum coagulation. *Environmental Science & Technology* **2003**, *37* (22), 5175-5180. DOI: 10.1021/es0343003.

(6) Mamane, H.; Shemer, H.; Linden, K. G. Inactivation of *E. coli, B. subtilis* spores, and MS2, T4, and T7 phage using UV/H_2O_2 advanced oxidation. *Journal of Hazardous materials* **2007**, *146* (3), 479-486. DOI: 10.1016/j.jhazmat.2007.04.050.

(7) Raza, S.; Folga, M.; Łoś, M.; Foltynowicz, Z.; Paczesny, J. The Effect of Zero-Valent Iron Nanoparticles (nZVI) on Bacteriophages. *Viruses* **2022**, *14* (5). DOI: 10.3390/v14050867.

(8) Nair, A. M.; Kumar, A.; Barbhuiya, N. H.; Singh, S. P. Electrochemical inactivation of enteric viruses MS2, T4, and Phi6 using doped laser-induced graphene electrodes and filters. *Environmental Science: Nano* **2023**, *10* (8), 2077-2089. DOI: 10.1039/d3en00124e.

(9) Dovbeshko, G. I.; Gridina, N. Y.; Kruglova, E. B.; Pashchuk, O. P. FTIR spectroscopy studies of nucleic acid damage. *Talanta* **2000**, *53* (1), 233-246. DOI: 10.1016/s0039-9140(00)00462-8.

(10) Movasaghi, Z.; Rehman, S.; Rehman, I. U. Fourier Transform Infrared (FTIR) spectroscopy of biological tissues. *Applied Spectroscopy Reviews* **2008**, *43* (2), 134-179. DOI: 10.1080/05704920701829043.

(11) Stuart, B. H. *Infrared Spectroscopy : Fundamentals and Applications*; John Wiley & Sons, Incorporated, 2004.

(12) Byler, D. M.; Susi, H. Examination of the secondary structure of proteins by deconvolved FTIR spectra. *Biopolymers* **1986**, *25* (3), 469-487. DOI: DOI 10.1002/bip.360250307.

(13) Barth, A. Infrared spectroscopy of proteins. *Biochimica et Biophysica Acta* **2007**, *1767* (9), 1073-1101. DOI: 10.1016/j.bbabio.2007.06.004.

(14) Jackson, M.; Mantsch, H. H. The use and misuse of FTIR spectroscopy in the determination of protein structure. *Critical Reviews in Biochemistry and Molecular Biology* **1995**, *30* (2), 95-120. DOI: 10.3109/10409239509085140.

(15) Zhang, K.; Young, R.; Zeng, L. Bacteriophage P1 does not show spatial preference when infecting *Escherichia coli*. *Virology* **2020**, *542*, 1-7. DOI: 10.1016/j.virol.2019.12.012.
